# Safety and Feasibility of a First-Person View, Full-Body Interaction Game for Telerehabilitation Post-Stroke

**DOI:** 10.5195/ijt.2018.6250

**Published:** 2018-08-03

**Authors:** RACHEL PROFFITT, JESSICA WARREN, BELINDA LANGE, CHIEN-YEN CHANG

**Affiliations:** 1 DEPARTMENT OF OCCUPATIONAL THERAPY, UNIVERSITY OF MISSOURI, COLUMBIA, MO, USA; 2 THE LAUNCHPAD THERAPY FOR KIDS, SAN JUAN CAPISTRANO, CA, USA; 3 PHYSIOTHERAPY, COLLEGE OF NURSING AND HEALTH SCIENCES, FLINDERS UNIVERSITY, ADELAIDE, AUSTRALIA; 4 INSTITUTE FOR CREATIVE TECHNOLOGIES, UNIVERSITY OF SOUTHERN CALIFORNIA, LOS ANGELES, CA, USA

**Keywords:** Head Mounted Displays, Stroke, Telerehabilitation, Virtual Reality

## Abstract

This study explored the feasibility and safety of pairing the Microsoft Kinect® sensor with the Oculus Rift® Head Mounted Display (HMD) as a telerehabilitation technology platform for persons post-stroke. To test initial safety, fourteen participants without disabilities (age 30 ± 8.8 years) engaged in a game-based task using the Microsoft Kinect® with a first-person view using the Oculus Rift®. These tasks were repeated for five participants post-stroke (age 56 ± 3.0 years). No significant adverse events occurred in either study population. When using the Oculus Rift® HMD, three participants without disabilities reported dizziness and nausea. All of the participants post-stroke required hands-on assistance for balance and fall prevention. The intensive nature of physical support necessary for this type of interaction limits the application as a telerehabilitation intervention. Given the increasing availability of HMDs for commercial use, it is crucial that the safety of immersive games and technologies for telerehabilitation is fully explored.

Interactive virtual reality (VR) tools are gaining acceptance and use in healthcare fields (Laver et al., 2017; Rizzo & Kim, 2005). Specifically, physical rehabilitation (including occupational therapy and physical therapy) has readily adopted movement-based VR tools such as the Nintendo Wii® and the Microsoft Kinect®. Both off-the-shelf and customized applications using these tools have had much success in improving rehabilitation client outcomes (Laver et al., 2017) and generally have good user acceptance (Lange, Flynn, & Rizzo, 2009). Within the rehabilitation field, many of these applications are targeted to people post-stroke (Laver et al., 2017). New technologies and software applications continue to be developed and utilized explicitly for persons post-stroke including virtual reality-based robotics, upper extremity exoskeletons, and Microsoft Kinect®- based games (Laver et al., 2017).

The Microsoft Kinect® as “controller” for a rehabilitation game can be readily accessed and used by persons post-stroke in both the clinic and home environment. We have developed software called *Mystic Isle* that utilizes the Microsoft Kinect® sensor as the input device (Lange et al., 2012). *Mystic Isle* was designed as a rehabilitation game and has shown good results in improving motor function and daily activity performance in persons post-stroke as a home exercise program (Proffitt & Lange, 2015). The *Mystic Isle* game involves multi-planar, full body movements. Designed for individuals with diverse abilities, games can be played in a sitting or standing position, depending on the therapy treatment plan. In the standing position, the player is able to move around in the three-dimensional (3D) space, akin to real-world rehabilitation. Players view an “avatar” of themselves on the screen from a third-person perspective. Most persons post-stroke generally enjoy this view of the game; however, some have expressed a desire to be able to more definitively navigate and view the 3D virtual space (Proffitt & Lange, 2015). A first-person view within a virtual environment may be able to address this need.

Head-mounted displays (HMDs) provide a first-person view of a virtual environment. One such HMD, the Oculus Rift®, provides a 120 degree view of the virtual environment with the ability for a user to look around 360 degrees within the space by turning his or her head. For persons post-stroke, HMDs have been employed as the viewer for some attention-based games and navigational tasks (Gamito et al., 2014). The player or user controls movement and selection within the virtual environment via a controller (e.g., joystick, Microsoft Xbox controller, phone buttons). Most of the research exploring the potential of HMDs for rehabilitation employs games that are static, seated (usually an exercise bike), or employs pieces of equipment that are available only in a laboratory or clinic setting (Gobron et al., 2015; Shaw et al., 2015; Yates, Keleman, & Lanyi, 2015). Even fewer systems are used with a telerehabilitation platform and involve games for cognitive rehabilitation or exercise on a stationary bike (Boulanger, Pournajib, Mott, & Schaeffer, 2017; Gamito et al., 2014). Head mounted displays are becoming more widespread in rehabilitation, and low-cost versions compatible with smartphones can be easily acquired by persons receiving rehabilitation services. It is imperative that clinicians (i.e., occupational therapists and physical therapists) have sufficient knowledge and training before utilizing these tools in clinical practice and as part of a telerehabilitation intervention.

We have paired the first-person viewpoint of the Oculus Rift® with the full-body tracking capabilities of the Microsoft Kinect®. In this pilot study, we conducted two consecutive experiments to explore the safety and feasibility of pairing the Oculus Rift® HMD in combination with the Microsoft Kinect® sensor as a full-body interactive experience for persons without disabilities and for persons more than six months post-stroke. Addressing the issues of safety and clinical feasibility, including acceptability and practicality, is necessary and prudent at this point in the research process (Bowen et al., 2009). The safety of the individual components (Oculus Rift® and Kinect®) have been investigated (Proffitt & Lange, 2015; Moss & Muth, 2011; Sharples, Cobb, Moody, & Wilson, 2008); however the combination has not been assessed.

## EXPERIMENT 1

### METHODS

#### PARTICIPANTS

Participants were recruited by a convenience sample from the University of Southern California. Participants were eligible for the study if they were (1) over the age of 18 years, (2) could read and understand English, and (3) had no medical condition for which they had been advised by a doctor to avoid watching television or playing video games. This portion of the study was approved by the Institutional Review Board at the University of Southern California. Written consent was obtained from all subjects prior to study enrollment.

#### STUDY DESIGN

This was a quasi-experimental, randomized crossover design. To minimize ordering effects, the order of interactions was counterbalanced across participants. Participants first completed a brief training interaction to become familiar with the Kinect® tracking. Participants completed the three tasks described above under two different experimental conditions, described below.

- Condition 1 (Figure 1a): The participants completed all tasks using the Microsoft Kinect® camera as the tracking device. Participants stood about 6–8 feet from the Kinect® camera. The virtual environment (recycling plant) was viewed on a 43” TV monitor. The participants viewed a semi-transparent avatar representation of themselves in the virtual environment. This avatar did not obstruct the participants’ view of the task.
- Condition 2 (Figure 1b): The participants completed all tasks using the Microsoft Kinect® camera as the tracking device. Participants stood about 6–8 feet from the Kinect® camera. Participants viewed the virtual environment using the Oculus Rift® DK2. The Oculus Rift® was tethered to the computer providing the virtual environment via a 10-foot long HDMI and USB cord. The participants’ view of the virtual environment was as though they were looking through the eyes of the avatar. By wearing the Oculus Rift® HMD, the participants were able to complete tasks from a first-person perspective in the recycling plant. For example, if the participants looked down, they would see the avatar representation of their torso, legs, and feet. If they held out their hands, they would see the avatar representation of their arms and hands.

#### SAFETY

Participants were told to stop if they felt dizzy, nauseous, off-balance, or had blurry vision, and such incidences were documented. A licensed and certified occupational therapist was available for balance stand-by assistance during the entire session. Participants were monitored for physiological changes such as increased respiration rate and flushing and/or whitening of the face and chest. If members of the research team noticed such a change, they queried the participant. Rest breaks were offered between conditions.

#### VIRTUAL ENVIRONMENT & TASKS

**Figure 2:**
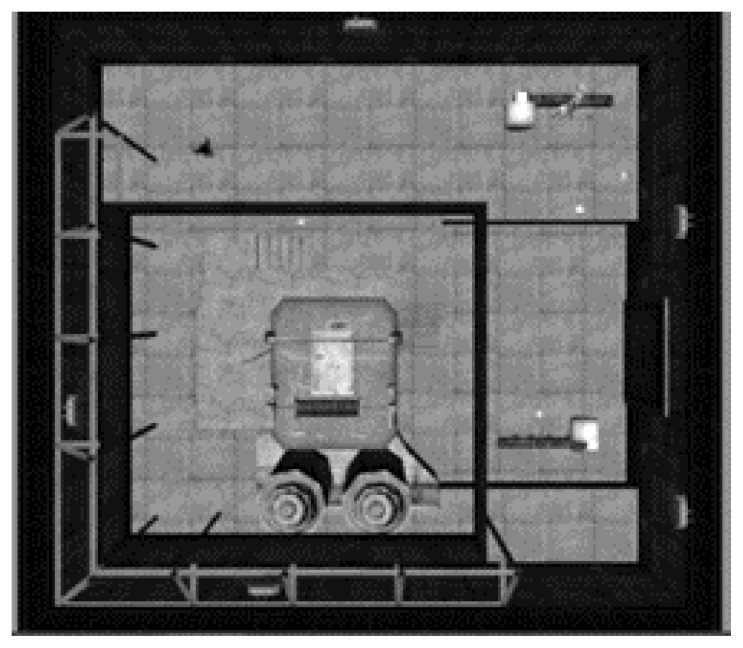
An overhead view of the recycling plant.

The virtual environment was a 3D recycling plant (Figure 2) in which the player completed three different tasks (described below) designed to simulate the real world. All three tasks and each experimental condition used the Microsoft Kinect® sensor (SDK V1.8) to track full body movements. The software was developed using Unity 3D Engine.

#### TASK 1: BOTTLE-SORTING

The player controlled an avatar to sort red, white, and green bottles from a conveyor belt into three color-matched bins on the floor (Figure 3a). Each correct sort within three minutes earned the player one “point.”

#### TASK 2: BOTTLE-FILLING

The player controlled an avatar to fill five bottles on a conveyor belt by pressing a button above each one (Figure 3b). A forward reach activated the button “press.” The bottles were all different heights; therefore, each bottle took a different amount of time to fill up. The player was instructed to fill up each bottle without overfilling it. Each successful “fill” within three minutes earned one “point.” Each “overfill” resulted in a one “point” deduction.

#### TASK 3: BAG-LOADING

The player controlled an avatar to “pick up” three garbage bags from the floor and place them, one at a time, in a mechanical hook at eye level (Figure 3c). Both hands were required to be used together to “pick up” the bags. Each bag successfully placed on the hook earned the player one “point.”

### OUTCOME MEASURES

All participants completed a demographic and a brief technology use questionnaire. At the completion of all experimental conditions, the participant answered semi-structured interview questions. All interviews were audio-recorded.

### DATA ANALYSIS

The questionnaire data were entered into a RedCap database; an independent researcher verified accuracy. Researchers applied descriptive statistics to the demographic data, and a content analysis to the interview data. Different researchers coded the interviews and the code list was continually refined. Broad themes and sub-themes emerged from the data and were refined as well.

## RESULTS

### PARTICIPANT DEMOGRAPHICS

Fourteen participants (3 males, 11 females) completed the evaluation. Participants ranged in age from 20–50 years (mean age 30 years ± 8.8). Most did not regularly play video games and none had interacted with a head mounted display (of any brand) before this study.

#### SAFETY

None of the participants had a loss of balance during either condition. Two participants needed to stop playing in Condition 2 due to dizziness before they had completed all three tasks. One of these two participants stopped at 1 minute 35 seconds into Task 3 and a second stopped at 2 minutes 4 seconds into Task 3. A third participant completed all three tasks but reported that she felt dizzy after removing the Oculus Rift®. A chair and water were offered to participants. All were able to complete the post-assessments and interview. There were no observable losses of balance or falls.

#### POINT OF VIEW

Most participants felt more engaged in the game when using the Oculus Rift® HMD as compared to the third person view. While using the Oculus Rift® HMD, participants were able to “see” and perceive the virtual environment better. Many of the participants described feeling that they were better able to coordinate movements and that their movements were more easily recognized by the technology than in the third-person view of the virtual environment. As one participant stated, “It was much easier to know where my body was in space and to see and to feel more competent when I was using the Oculus.”

### PRELIMINARY DISCUSSION

The preliminary safety data from Experiment 1 informed our decision to move forward with Experiment 2 and test the pairing of the Kinect® and the Oculus Rift® HMD in persons post-stroke. With only three people reporting dizziness and no falls or adverse events, the Kinect® and Oculus Rift® HMD can be tested in rehabilitation populations. The recycling plant games used in Experiment 1 were designed solely for that purpose and have no rehabilitation purpose. Further, the recycling plant cannot be customized to the diverse motor abilities of persons post-stroke. Therefore, we sought to further explore the safety and feasibility of the Oculus Rift® HMD and Kinect® pairing with persons post-stroke using a customized rehabilitation game called *Mystic Isle*.

## EXPERIMENT 2

### METHODS

#### PARTICIPANTS

Participants were recruited by a convenience sample from the University of Missouri. Participants were eligible for the study if they: (1) were over the age of 18 years, (2) were at least 6 months post-stroke, with a score between 6 and 20 on the NIH Stroke Scale (mild-moderate stroke), (3) were able to follow 2-step directions, (4) were able to understand conversational English, and (5) were not under the advice of a physician to avoid watching television or playing video games. This portion of the study was approved by the University of Missouri Health Sciences Institutional Review Board. Written consent was obtained from all subjects prior to study enrollment.

#### STUDY DESIGN AND SAFETY

Participants completed the same two experimental conditions described in Experiment 1. They were instructed to stop if they felt dizzy, nauseous, off-balance, or had blurry vision. A licensed and certified occupational therapist provided hands-on assistance for balance for all participants during Condition 2. Participants were monitored for physiological changes such as increased respiration rate and flushing and/or whitening of the face and chest. If members of the research team noticed such a change, they queried the participant. The number of times participants reported any of the above changes or felt off balance were recorded. Participants were offered rest breaks between conditions.

#### VIRTUAL ENVIRONMENT & TASKS

The virtual environment, called *Mystic Isle,* was developed using Unity 3D Engine. This game environment was previously described (Lange et al., 2012; Proffitt & Lange, 2015). The games used the Microsoft Kinect® sensor to track full body movements of the participant. The locations of the virtual objects were calibrated to each participant’s extent of reach in both arms. The participant played two games, described below.

#### GAME 1: SIMPLE REACHING

The participant controlled an avatar to reach out and touch a virtual object that was lit up red (Figure 4a). The participant “touched” 16 virtual objects.

#### GAME 2: SORTING

The participant controlled an avatar to “sort” colored objects into colored areas in the virtual environment (Figure 4b). The participant selected an object and then moved it across the screen to the corresponding colored area. The participant sorted 16 objects.

### OUTCOME MEASURES

Participants completed the same outcome measures as in Experiment 1. Three participants had expressive aphasia and mild cognitive deficits and were unable to complete the semi-structured interview. They were able to answer yes/no questions in place of an interview.

### DATA ANALYSIS

Data were recorded as in Experiment 1, and descriptive statistics calculated for the demographic data. Given the small sample size, the interview data were summarized.

## RESULTS

### PARTICIPANT DEMOGRAPHICS

Five participants (3 males, 2 females) completed the evaluation. Participants ranged in age from 53 to 59 years (mean age 56 years ± 3.0). Participants did not regularly play video games and none had interacted with a head mounted display (of any brand) before this study.

### SAFETY

None of the participants reported feeling dizzy or nauseous during or after the experiment. One participant noticeably squinted throughout the interview and reported that she had some pain in her left eye, a regular occurrence since her stroke. The researchers asked her to inform both the study team and her doctor if the pain increased. She did not contact the researchers after the study concluded.

All of the participants required hands-on balance assistance during the Oculus HMD + Kinect Condition. Three participants required hands-on assistance to avoid falling while stepping forward. Four of the five participants required assistance when reaching out of the base of support (forward or side). No falls occurred. Twenty-three instances of loss of balance occurred for all five participants; these required hands-on assistance.

### USABILITY IN THE STROKE POPULATION

Two of the five participants preferred the third person view of the game. Participants felt that they “… could see [the targets] better” and “[the game] was easier to process” when viewing the virtual environment and the avatar on the screen. The two participants who preferred the third person view had limitations in their visual perception abilities and referenced that limitation when stating the preference. All participants felt that either method of delivery would be beneficial as a rehabilitation game. For example, one participant felt that because people spend so much time using electronic devices, “… it would absolutely be beneficial to turn the play into something constructive.”

### DISCUSSION

The purpose of this study was to explore the safety and feasibility of pairing the Oculus Rift® HMD in combination with the Microsoft Kinect® sensor as a full-body interactive experience for persons without disabilities and for persons post-stroke. Most of the previous research on HMDs in persons post-stroke have been keyboard/mouse/joystick controller-based systems (Kronqvist, Jokinen, & Rousi, 2016). Games that require players to use their body as the controller are inherently distinct and warrant investigation. To our knowledge, this was the first study to explore the safety and feasibility of pairing a HMD with a full-body interactive rehabilitation game.

People without disabilities preferred the first-person view of the world and felt more in control. For the five persons post-stroke, there was not a clear preference. Despite the small sample, it seemed as though visual perception played a role in participant preference. With the ultimate goal of designing a safe and feasible rehabilitation game, we must also consider other factors such as cost and time (Cox, Cairns, Shah, & Carroll, 2012; Kizony & Katz, 2003). Therefore, if a seemingly more immersive method of game play does not lead to an increased sense of presence for people with disabilities, we must be cautious moving forward in future studies.

A few participants without disabilities (about 20%) stated that when the task became too difficult, they lost that sense of “being there” in the virtual environment. For training or rehabilitation games, the goal is to customize tasks so that the difficulty level is at a “just-right” level of challenge (Profitt & Lange, 2015). Others have noted the role that task difficulty plays in immersion and presence (Cox et al., 2012; Schrader & Bastiaens, 2012). Moving forward, a customized set of tasks for each participant may be necessary, especially when the participant is completing movement-based tasks. The height of the player and motor skill ability can impact task success and need to be taken into consideration when developing the player’s profile and games.

No significant adverse events occurred during this study. In Experiment 1, only three people reported any symptoms of nausea; of those two had to stop early. Both stopped during Task 3, the bag-loading task. This task involves large movements such as bending and squatting. Although there is no perceptible lag in the HMD, there is no gaze stabilization. For example, when participants wearing the Oculus® turns their head, their gaze in the virtual world follows the movement of their head. It is impossible for a person to keep his or her gaze locked on a virtual target and move the head, a task that is achievable by persons without disability. This is usually the cause of “simulator sickness” in virtual environments and is well documented in prior research (Moss & Muth, 2011; Sharples et al., 2008). The persons post-stroke did not do any bending or squatting and thus the HMD may not have triggered the same reaction.

We have safely utilized the third-person view of the *Mystic Isle* game with people with stroke as a telerehabilitation intervention (Proffitt & Lange, 2015). There were no reported adverse events or falls reported in that study. In addition, we ensured that each study participant had appropriate safety measures in place such as caregiver oversight, a sturdy chair, or a cane (Proffitt & Lange, 2015). For this study, all of the persons post-stroke required some form of hands-on assistance for safety and observed losses of balance. The addition of the Oculus Rift® may require safety supports beyond what is feasible in a home or community setting. The intensive nature of physical support necessary for this type of interaction limits the application as a telerehabilitation intervention. We provide a word of caution to rehabilitation providers seeking to use these kinds of technologies for telerehabilitation and similarly suggest that they carefully advise clients who seek to use them on their own.

This study has a number of limitations. First, it was a one-time study with a short time frame. Therefore, it is difficult to translate these findings to long-term use and gameplay. Secondly, this was a small sample size, with diverse participants. This makes it difficult to generalize the findings to other populations. Third, the movements the persons post-stroke performed to play the game were limited in scope. Further assessments of safety with those who are able (e.g., participants after a mild stroke) to perform large movements are needed.

A range of possibilities exist for future research stemming from this pilot study. For example, this combination of technologies could be explored with other people with disabilities, such as people who have experienced a spinal cord injury or a traumatic brain injury. HMDs are becoming increasingly ubiquitous in both clinical and non-clinical settings. This is one example of a combination of technologies that we believe can be applied to other commercially available HMDs that have integrated sensors and controllers. Lastly, most of the research using games for training and rehabilitation has focused on the efficacy of the games in improving clinical outcomes (Laver et al., 2017). Few studies have considered the impact of player point-of-view on performance, enjoyment, and presence. It is critical that we elucidate the underlying “active ingredients” in virtual reality-based games for training and rehabilitation so that we can design the most impactful telerehabilitation interventions.

## Figures and Tables

**Figure 1a:**
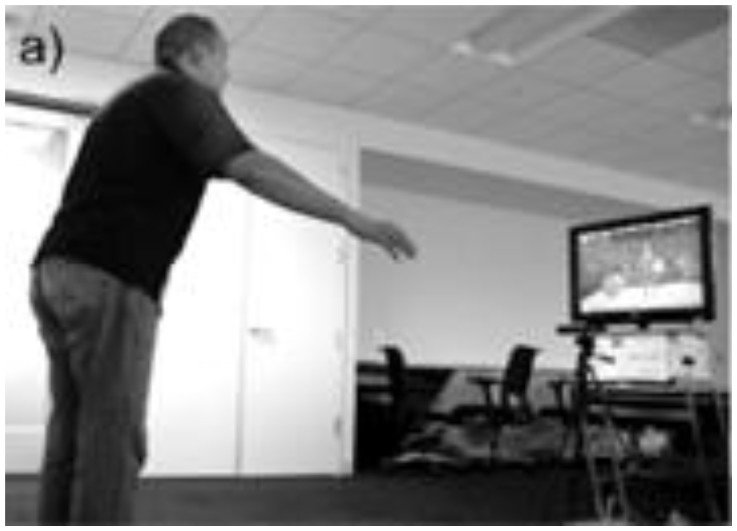
The third-person view of the game. The participant views the game and avatar on a TV monitor.

**Figure 1b:**
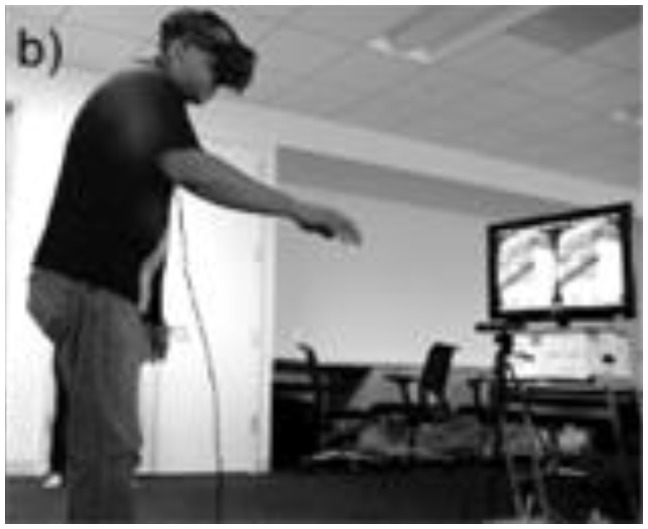
The first-person view of the game. The participant views the game through the Oculus Rift® HMD. The view on the monitor is parsed out by the individual lenses.

**Figure 3a:**
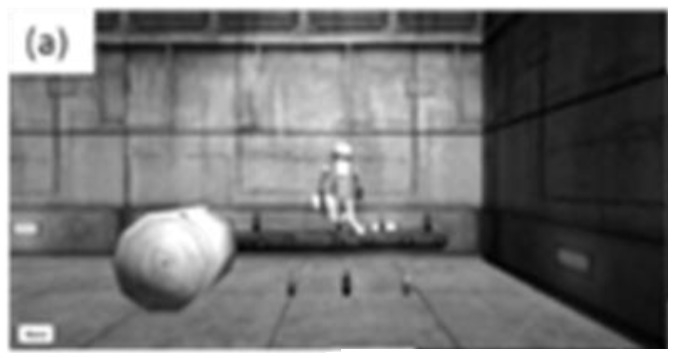
The bottle-sorting task: The three bottles on the ground indicate to the player where to place the bottles for sorting.

**Figure 3b:**
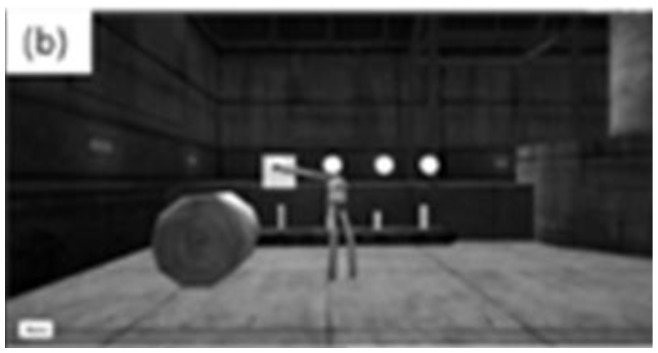
The bottle-filling task: The player holds out a hand “over” the button to “fill up” the bottle.

**Figure 3c:**
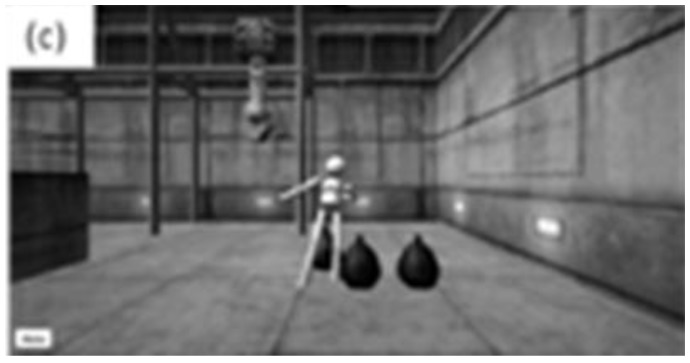
The bag-loading task: The player “picks up” the bag to place it on the hook in the background.

**Figure 4a:**
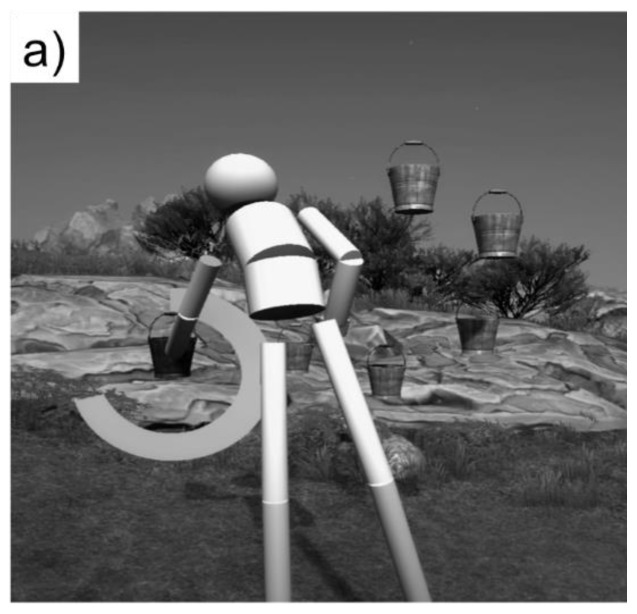
The simple reaching task.

**Figure 4b:**
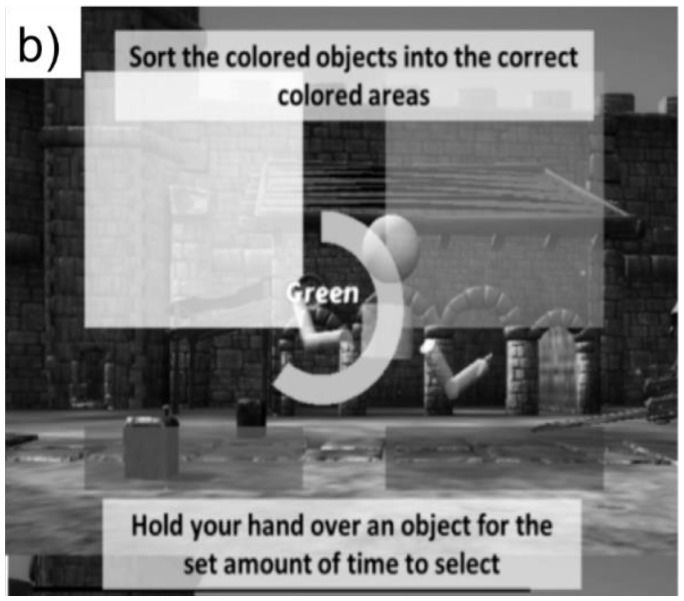
The sorting task.
